# Measuring the Humoral Immune Response in Cats Exposed to Feline Leukaemia Virus

**DOI:** 10.3390/v13030428

**Published:** 2021-03-07

**Authors:** Yasmin A. Parr, Melissa J. Beall, Julie K. Levy, Michael McDonald, Natascha T. Hamman, Brian J. Willett, Margaret J. Hosie

**Affiliations:** 1 MRC—University of Glasgow Centre for Virus Research, Glasgow, Scotland G61 1QH, UK; Brian.Willett@glasgow.ac.uk (B.J.W.); Margaret.Hosie@glasgow.ac.uk (M.J.H.); 2 IDEXX Laboratories, Inc.—Westbrook, ME 04092, USA; Melissa-Beall@IDEXX.com; 3 Maddie’s Shelter Medicine Program, University of Florida, Gainesville, FL 32608, USA; levyjk@ufl.edu; 4 Veterinary Diagnostic Services, University of Glasgow, Glasgow, Scotland G61 1QH, UK; Mike.McDonald@glasgow.ac.uk; 5 Austin Pets Alive!, Austin, TX 78703, USA; nth4@utexas.edu

**Keywords:** FeLV, retrovirus, humoral immune response, diagnostics, exposure outcomes, SU antibody response

## Abstract

Retroviruses belong to an important and diverse family of RNA viruses capable of causing neoplastic disease in their hosts. Feline leukaemia virus (FeLV) is a gammaretrovirus that infects domestic and wild cats, causing immunodeficiency, cytopenia and neoplasia in progressively infected cats. The outcome of FeLV infection is influenced by the host immune response; progressively infected cats demonstrate weaker immune responses compared to regressively infected cats. In this study, humoral immune responses were examined in 180 samples collected from 123 domestic cats that had been naturally exposed to FeLV, using a novel ELISA to measure antibodies recognizing the FeLV surface unit (SU) glycoprotein in plasma samples. A correlation was demonstrated between the strength of the humoral immune response to the SU protein and the outcome of exposure. Cats with regressive infection demonstrated higher antibody responses to the SU protein compared to cats belonging to other outcome groups, and samples from cats with regressive infection contained virus neutralising antibodies. These results demonstrate that an ELISA that assesses the humoral response to FeLV SU complements the use of viral diagnostic tests to define the outcome of exposure to FeLV. Together these tests could allow the rapid identification of regressively infected cats that are unlikely to develop FeLV-related disease.

## 1. Introduction

**Figure 1:**
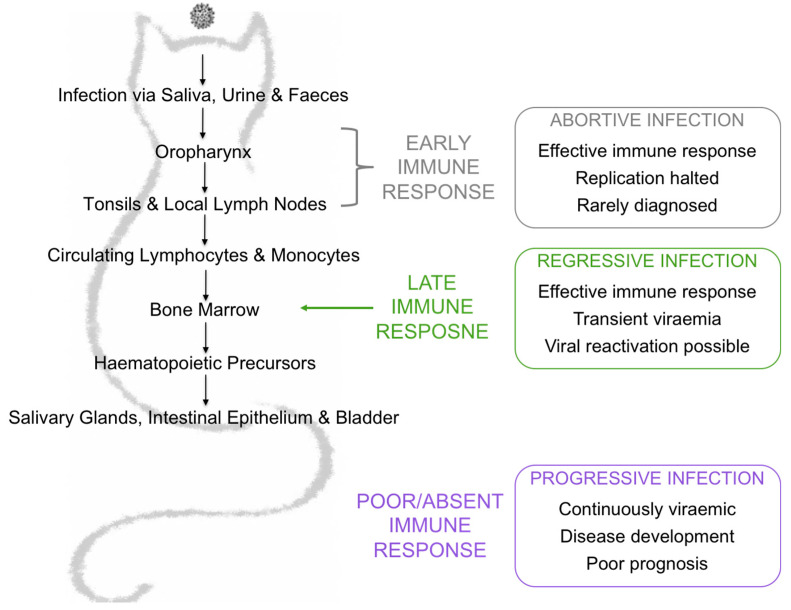
Exposure outcomes are largely influenced by the immune response to infection. Abortive infection is the result of a low dose exposure to FeLV or an effective and specific immune response early in infection. Regressive infection is the result of an effective immune response later in infection, either just before or just after bone marrow infiltration. Cats with regressive infection are transiently antigenaemic and viraemic. After weeks to months, the viraemia is controlled and these cats become aviraemic. Progressive infection is the result of a poor immune response. Cats with progressive infection are continuously viraemic and have a poor prognosis, developing FeLV-associated diseases and usually have a limited lifespan.

FeLV is a single-stranded, positive-sense RNA gammaretrovirus that has global impact, infecting domestic and wild felids worldwide. FeLV can be isolated from the saliva, urine and faeces of viraemic cats and activities such as grooming, fighting and the shared use of food bowls and litter trays facilitates horizontal transmission via the oronasal route [1,2,3,4]. The prevalence of FeLV has decreased greatly as a result of effective vaccination and identification and segregation of infected cats, but remains high (ranging from 5 to 20%) in at-risk groups such as sick cats and cats from multi-cat households [5,6]. FeLVs exist as a group of viruses distinguished by their SU glycoprotein gene sequences, which influence receptor usage, tissue tropism and, ultimately, disease outcome. FeLV-A is thought to be the predominant transmissible form of FeLV, whereas subgroups FeLV-B and FeLV-C arise de novo in FeLV-A-infected cats following recombination with endogenous FeLV sequences and mutation, respectively. However, there is some evidence to suggest that these endogenous variants could be transmitted between cats [7]. The outcome following FeLV exposure is complex, unpredictable and dependent on many factors, including the route of infection, the age of the host at the time of infection, concurrent co-infections, stress and the dose of virus to which the host was exposed [8,9,10,11]. Cats that become infected following FeLV exposure can develop abortive, regressive or progressive infections, depending on their immune response to infection. (Figure 1).

Abortive infection has been documented in naïve cats experimentally challenged with a low dose of FeLV as well as in experimentally challenged, FeLV-vaccinated cats [12,13]. Viral replication is rapidly halted and cats with such infections are seldom identified. Cats with regressive infection typically endure a short viraemia before viral replication is suppressed. Although regressively infected cats are typically neither antigenaemic nor viraemic, they do harbour virus in their bone marrow. However, they do not pose any threat to naïve cats, unless viral reactivation occurs due to severe stress or immunosuppression. Progressive infection occurs when the viral load overcomes the ability of the immune response to eliminate the virus and the cat is both antigenaemic and viraemic. Such cats ultimately develop FeLV-associated diseases such as leukaemia, lymphoma and non-regenerative anaemia. Since progressively infected cats are persistently viraemic, they act as a viral reservoir and source of infection for other cats. Cats with progressive infection have a poor prognosis, with an average lifespan of 3 years post infection [3,14].

Focal infections are rarely documented in nature but have been reported in up to 10% of experimental infections [15]. Focal infections are associated with atypical local infection in tissues such as lymph nodes, spleen, mammary glands, bladder and eyes. Viral replication in infected tissues leads to the production of p27 capsid antigen that causes intermittent, weak antigen positive results. In addition, the infected tissues contain proviral DNA that is absent from the bone marrow and blood [4,16]. Cats display different diagnostic profiles depending on their stage of infection and subsequent exposure outcome, as shown in Table 1.

It is recommended that samples from cats that test positive for p27 antigen using in-clinic point of care tests should undergo confirmatory testing by a diagnostic laboratory [17,18]. Cats that are confirmed antigen positive are then tested for proviral DNA using polymerase chain reaction (PCR)-based tests. Viraemic cats may also be identified using plasma virus isolation, although this test is not widely available. It is advised that viraemic cats are isolated from other cats and retested six weeks later, enabling the differentiation of cats with regressive and progressive infection [5,18]. Cats can be described as discordant when they test positive for certain diagnostic markers but negative for others.

The antibody response to FeLV infection is directed mainly towards the viral surface unit (SU) glycoprotein (gp70) and p15E [10], although antibodies that recognise all major structural proteins have been detected in sera of cats with regressive infection [19]. Cats that develop virus neutralising antibodies (VNA) are protected from infection following exposure to the virus [20] and kittens that passively receive maternally derived antibodies are transiently protected from infection [10,21]. Most cats with regressive infection demonstrate high VNA titres; a high VNA titre is a good indicator of virus suppression such that clinical disease does not develop [22,23]. Therefore, an accurate and time-efficient measurement of an appropriate, specific antibody response would allow clinicians to stratify FeLV-infected animals more rapidly. Serological testing has the potential to aid the identification of cats with regressive infection and provides a means of monitoring the immune response to infection. While the immune response is maintained, the likelihood of viral reactivation remains low. Therefore, the aim of this study was to assess the utility of an enzyme-linked immunosorbent assay (ELISA) measuring the humoral immune response to FeLV SU to predict the outcome of FeLV exposure, testing sequential samples collected from shelter cats at high risk of FeLV infection.

## 2. Materials and Methods

### 2.1. Samples

Cats were enrolled in the study if they tested positive for p27 capsid antigen using anticoagulated whole blood on the IDEXX SNAP^®^ FIV/FeLV Combo Test (IDEXX Laboratories, Inc., Westbrook, ME, USA) when screened on admission to the Austin Pets Alive! Shelter (Austin, TX, USA). These cats remained in the study regardless of subsequent test results. In total, 123 cats were included in this study. Heparinised whole blood samples were received from 66 cats and paired samples of heparinised whole blood, collected at least one month apart, were received from 57 of these cats. The majority of paired samples were collected 6 months apart, as shown in Table 2.

Plasma was separated from heparinised whole blood by centrifugation (200× *g* for 5 min). Peripheral blood mononuclear cells (PBMC) were then isolated from the remaining fractions by density gradient centrifugation using Ficoll-Paque Plus (GE Healthcare Life Sciences, Manchester, UK).

### 2.2. Detection of Plasma Antigenaemia

Plasma antigenaemia was determined at IDEXX Laboratories (Westbrook, ME, USA) by testing plasma samples for p27 capsid antigen using the IDEXX FeLV PetChek^®^ ELISA. All positive results were confirmed using the PetChek^®^ ELISA neutralisation protocol as per the manufacturer’s instructions [24]. Quantitative results were determined using a standard curve as outlined previously [25]. In this instance, 30 ng/mL was the upper limit of quantitation.

### 2.3. Virus Isolation from PBMC

Following separation from whole blood samples, PBMC were cultured with Concanavalin A (5 µg/mL, Sigma-Aldrich, Kent, UK) for 21 days in RPMI medium (Gibco^™^, Renfrew, UK) supplemented with 10% foetal bovine serum (Hyclone, ThermoFisher Scientific, Renfrew, UK), 2 mM L-glutamine (Invitrogen^™^, Renfrew, UK), 100 U/mL penicillin (Invitrogen^™^), 100 µg/mL streptomycin (Invitrogen^™^), 50 µM 2-mercaptoethanol (Sigma-Aldrich) and 100 IU/mL interleukin 2. Culture fluids were sampled on day 14 (D14) and day 21 (D21) and the media were changed on D14. Culture fluids were centrifuged to remove cells and samples of supernatant were stored at −80 °C until required. After 21 days in culture, PBMC were stored at −80 °C until required for DNA extraction and subsequent proviral DNA detection.

### 2.4. Detection of Reverse Transcriptase in PBMC Culture Fluids

Reverse transcriptase (RT) activity in D14 and D21 PBMC culture fluids was measured using a product enhanced reverse transcriptase (PERT) assay, based on previously described methods [26,27]. Briefly, 10 µL of culture fluid were added to an equal volume of lysis buffer (containing 50 mM KCL, 0.1 mM Tris pH 7.4, 40% glycerol, 0.25% Triton-X-100) and incubated at room temperature. After 10 min, 80 µL of nuclease-free water were added to give a final sample dilution of 1:10. To each well of a 96 well MicroAmp^®^ Fast reaction plate (Applied Biosystems^®^), 5 µL of lysed, diluted sample were added to 15 µL of master mix containing TaqMan Universal PCR Master Mix (Applied Biosystems^®^), 2 mg/mL bovine serum albumin (BSA, New England Biolabs^®^), 40 U/µL RNasin ribonuclease inhibitor (Promega, Southampton, UK), 0.8 µg/µL MS2 phage RNA (Roche, Burgess Hill, UK), 10 mg/mL calf thymus DNA (Invitrogen^™^), 20 pmol/µL MS2 phage forward primer (5′-GCC TTT CTC ATT CGT TGT CG-3′), 20 pmol/µL MS2 phage reverse primer (5′-GCT TAT GAT GGA CTC ACC CG-3′) and 5 pmol/µL fluorescent PERT probe (5′(FAM)-TCT TTA GCG AGA CGC TAC CAT GGC TA-(TAMRA)3′). Calf thymus DNA was added to the PERT reaction to remove non-specific activity, a well-recognised method that suppresses endogenous DNA polymerase activity [28]. Murine leukaemia virus (MuLV) RT (New England Biolabs^®^) was used as a positive control and a standard curve was constructed, covering 100 U/mL to 0.001 U/mL of RT. RPMI medium was used as a negative control. MuLV RT standards did not require lysis prior to testing, therefore 5 µL of each standard were added to 15 µL of master mix. Amplification steps were carried out in an ABI PRISM^™^ 7500 Sequence Detection System (Applied Biosystems^®^) using the following parameters: 48 °C for 30 min for reverse transcription, 95 °C for 10 min to activate the DNA polymerase followed by 40 cycles of 95 °C for 15 s (denaturation) and 60 °C for 1 min (annealing and extension). Primers and probe were designed to amplify and detect MS2 phage DNA. If RT was present in the sample, MS2 phage RNA was reverse transcribed into MS2 phage DNA and then amplified and detected by the primers and probe. Absolute quantities of RT were not calculated. The standard curve was utilised as a positive control and to assess assay variation. All samples were tested in triplicate and average Ct values were calculated. Samples that tested negative for RT were recorded as having Ct values of 40.

### 2.5. Detection of p27 Capsid Antigen in PBMC Culture Fluids

The IDEXX FeLV PetChek^®^ ELISA was used to measure p27 capsid antigen in D14 and D21 PBMC culture fluids. All D21 culture fluids that tested positive were confirmed by the PetChek^®^ ELISA neutralisation protocol as per the manufacturer’s instructions [24]. Any p27 capsid antigen positive D14 culture fluids where corresponding D21 culture fluids tested negative were also confirmed using the PetChek^®^ ELISA neutralisation protocol. All plates were read at 650 nm to determine absorbance (A_650_).

### 2.6. Detection of FeLV Proviral DNA in PBMC

Proviral DNA was detected by qPCR on PBMC DNA extracted after 21 days of culture *in vitro*. DNA was extracted from PBMC cell pellets using the DNeasy^®^ Blood & Tissue Kit (QIAGEN^®^, Manchester, UK) as per the manufacturer’s instructions. The DNA concentration was determined spectrophotometrically using a NanoDrop^™^ spectrophotometer (ThermoFisher Scientific) and diluted to a final concentration of 25 ng/µL in nuclease-free water. Samples were tested in triplicate by qPCR using a protocol adapted from Cattori et al. [29] to detect FeLV proviral DNA and the housekeeping gene encoding feline β actin simultaneously. Briefly, to each well of a 96 well MicroAmp^®^ Fast reaction plate (Applied Biosystems^®^, Renfrew, UK), 4 µL of sample were added to 16 µL of master mix containing nuclease-free water and TaqMan universal PCR MM (Applied Biosystems^®^), 20 pmol/µL FeLV forward primer (5′-AACAGCAGAAGTTTCAAGGCC-3′), 20 pmol/µL FeLV reverse primer (5′- TTATAGCAGAAAGCGCGCG-3′), 6 pmol/µL FeLV probe (5′(FAM)-CCAGCAGTCTCCAGGCTCCCCA-(BHQ)3′), 1 pmol/µL feline β actin forward primer (5′- GACTACCTCATGAAGATCCTCACG -3′), 1 pmol/µL feline β actin reverse primer (5′- CCTTGATGTCACGCACAATTTCC-3′), 1 pmol/µL feline β actin probe (5′(YY)- CAGTTTCACCACCACCGCCGAGC-(BHQ)3′). The master mix preparation for FeLV standards also contained 100 ng per reaction of feline DNA extracted from QN10 cell line (a feline embryonic cell line). Primers and probes were purchased from IDT^®^ (Leuven, Belgium). Amplification steps were carried out in an ABI PRISM^™^ 7500 Sequence Detection System (Applied Biosystems^®^) using the following parameters: 50 °C for 2 min, 95 °C for 10 min, followed by 40 cycles of 95 °C for 15 s and 60 °C for 1 min. A puc18 plasmid containing the full genome of FeLV was used to construct a standard curve. In a background of 100 ng of DNA, 10 copies of FeLV were detectable in all three replicates and 1 copy of FeLV was detectable in at least one of three replicates. Proviral load (PVL) values were calculated for each sample from the standard curve that was performed on every plate.

### 2.7. Live Virus Neutralisation Assay

Live virus neutralising antibody assays were performed by Veterinary Diagnostic Services, University of Glasgow using the method based on the susceptible cell line QN10 [30]. Briefly, cells were seeded at 5 × 10^4^ cells/mL with 4 µg/mL polybrene (Sigma-Aldrich) and incubated for 24 h before serial dilutions of heat inactivated plasma samples (from 1:4 to 1:32) were incubated with a fixed titre of FeLV-A (Glasgow-1) and added to the cells. In the absence of neutralising antibodies, the FeLV virus induced plaque formation that was visible under light microscopy. The presence of VNA prevented infection of the cells and resulted in the preservation of an intact monolayer. Plates were examined after 4 days and the highest dilution containing a 75% plaque reduction compared to the virus control was taken as the VNA titre. Samples showing more than 75% plaque reduction at the 1:32 dilution were determined to have VNA titres ≥1:32.

### 2.8. Preparation of FeLV-A Immunoblots

The F422 cell line, a feline lymphoma cell line which stably expresses FeLV-A [31] was cultured in RPMI media supplemented with 10% foetal bovine serum (Hyclone, ThermoFisher Scientific), 2 mM L-glutamine (Invitrogen^™^), 100 U/mL penicillin (Invitrogen^™^), 100 µg/mL streptomycin (Invitrogen^™^), 50 µM 2-mercaptoethanol (Sigma-Aldrich) and 100 IU/mL interleukin 2. Culture fluids were collected from confluent cultures of F422 cells then filtered using a 0.45 µM filter. Next, sucrose gradient ultracentrifugation was performed. Briefly, a total of 35 mL of culture fluid was layered over 15 mL of 20% sucrose in thin-walled, ultraclear^™^ SW28 tubes (Beckman Coulter, Bromley, UK) and centrifuged using a Sorvall WX Ultra 100 ultracentrifuge (ThermoFisher Scientific) at 116,000× *g* for 2 h at 4 °C in a Surespin SW28 rotor. The supernatant was discarded, and the viral pellet was resuspended in 500 µL of bromophenol blue loading buffer. A total of 50 µL of pelleted F422 virus in bromophenol blue loading buffer was diluted in 50 µL of nuclease-free water and a further 50 µL of bromophenol blue loading buffer, then heated to 95 °C for 5 min and separated by electrophoresis using a single well (4–12%) precast polyacrylamide gel (Invitrogen^™^) at 100 volts for 2 h in MES buffer (New England Biolabs^®^, Hitchin, UK). Viral proteins were transferred to nitrocellulose using Trans-Blot^®^ Turbo^™^ (Bio-Rad, Watford, UK) and the membranes were blocked overnight in 1× casein buffer made using 0.1% phosphate buffered saline (PBS) Tween-20. The membranes were washed using 0.1% PBS Tween-20, dried and frozen at −20 °C until required.

### 2.9. FeLV-A Immunoblot Analysis

FeLV-A nitrocellulose membranes were thawed at room temperature and cut into strips of an appropriate width. Each strip was individually probed with either cat plasma or control antibody. Plasma samples were diluted 1:1000 using 1× casein buffer made using 0.1% PBS Tween-20. The murine monoclonal antibody VPG 19.1 [7] recognising FeLV p27 capsid antigen (used unpurified as hybridoma culture supernatant) was diluted 1:50 in casein buffer and the purified murine anti-SU monoclonal antibody was diluted to 1:1×10^6^ in casein buffer. Control and plasma samples were incubated with membrane strips for 1 h. The membrane strips were then washed, and biotinylated goat anti-cat IgG Fc (Vector Laboratories, Peterborough, UK) was diluted 1:1000 in casein buffer and incubated with the plasma sample incubated membranes for one hour. Biotinylated horse anti-mouse IgG Fc (Vector Laboratories) was diluted to 1:1000 in casein buffer and incubated with the VPG 19.1 and the purified anti-SU monoclonal antibody incubated membranes for 1 h. The membranes were washed and VECTASTAIN^®^ ABC-Amp reagent (Vector Laboratories) was added for 20 min as per the manufacturer’s instructions. The membranes were washed and the BCIP/NBT kit (a chromogenic alkaline phosphatase substrate, Vector Laboratories) was used as per the manufacturer’s instructions. The membranes were then washed for a final time. All wash steps were performed using 0.1% PBS Tween-20 and all incubation steps were performed at room temperature on an orbital shaker.

### 2.10. Production of SU Fusion Proteins

FeLV-A and FeLV-B SU-Fc fusion proteins were produced following the stable transfection of HEK293 cells with the respective FeLV SU in the pTORSTEN expression vector, using polyethylenimine (Polysciences Inc., Warrington, PA, USA), resulting in the expression of soluble FeLV SU bound to the C-terminal human IgG-Fc tag [7,32]. Transfected cells were selected using hygromycin B (Invitrogen^™^) at an initial concentration of 400 µg/mL, followed by 200 µg/mL for maintenance. Culture fluids containing the FeLV SU-Fc fusion proteins were harvested, filtered through 0.45 µm and 0.2 µm filters and frozen at −80 °C until required. Once 1 litre of culture fluid had been collected, the fusion protein was purified using HiTrap protein A sepharose columns (GE Healthcare Life Sciences). The bound protein was eluted from the column using 0.1 M sodium citrate pH 3.0 buffer and collected into fifteen 1.5 mL eppendorf tubes, each containing 350 µL of 1.5 M tris pH 8.6 to neutralise the fractions. The fractions containing protein were identified using the Bradford protein assay [33] and the positive fractions were pooled and dialysed against PBS pH 7.4 for 24 h. Protein expression was confirmed by immunoblotting using goat anti-Fc antibody (1:1000 dilution, Vector Laboratories) and murine anti-FeLV gp70 monoclonal antibody [7] (1:1×10^6^ dilution) and protein purity was assessed by Coomassie blue staining. Protein concentrations were determined using the Bradford protein assay [33] and serial dilutions of BSA (New England Biolabs^®^) were used to construct a standard curve.

### 2.11. Testing Plasma Samples for Reactivity against FeLV-SU

ELISA plates (Immulon^™^ 2HB high binding, ThermoFisher Scientific) were coated with FeLV-A or FeLV-B SU in coating buffer (100 mM sodium bicarbonate & 33 mM sodium carbonate anhydrous) at 100 ng/well in a final volume of 100 µL/well and incubated overnight on an orbital shaker at 4 °C. ELISA plates were then washed with 0.1% PBS Tween-20 and blocked in 1× casein buffer (200 µL/well, Vector Laboratories) prepared using Milli-Q water. After a one-hour incubation, plates were washed, plasma samples were diluted 1:200 in casein buffer and tested in triplicate (100 µL/well). Following the next one-hour incubation, plates were washed, and biotinylated anti-cat IgG (Vector Laboratories) was added (1:4000 in casein buffer, 100 µL/well) and incubated for one hour. Plates were washed and streptavidin horseradish-peroxidase (Vector Laboratories) was added (1:4000 in casein buffer, 100 µL/well) and incubated for 20 min. The plates were washed for a final time before tetramethylbenzidine (TMB) substrate (Sigma Aldrich) was added (100 µL/well), the plates were incubated for 15 min and then 1M sodium hydroxide was added to stop the reaction (50 µL/well). All wash steps were performed using 0.1% PBS Tween-20 and the incubations were performed at room temperature. Plates were read using a Multiskan Ascent^™^ microplate reader (ThermoFisher Scientific) at 420 nm to determine absorbance (A_420_). As each sample was tested in triplicate, average A_420_ values were calculated. A pool of cat plasma samples from experimentally infected cats known to have high neutralising antibody titres (≥1:128) was used as a positive control and a pool of plasma samples from cats with low reactivity to FeLV-A by immunoblot was used as a negative control. Normalised A_420_ values ([sample A_420_—negative control A_420_]/[positive control A_420_—negative control A_420_]) and average A_420_ values were calculated using Microsoft Excel (version 16.16.15).

### 2.12. Statistics

RStudio^©^ (version 1.2.1335) was used to determine the distribution of the data using the Shapiro Wilk Normality test, demonstrating that all data were distributed non-parametrically. All other statistical analyses were performed using Prism software (version 9.0, GraphPad Software, CA, USA). Two tailed Mann–Whitney tests were used to compare unpaired group mean values and the Wilcoxon matched-pairs signed rank test was used to compare paired sample mean values. The Kruskal–Wallis test and Dunn’s multiple comparisons tests were used to compare multiple group means. *p* values less than 0.05 were regarded as statistically significant.

## 3. Results

### 3.1. Production of FeLV-SU Proteins

**Figure 2:**
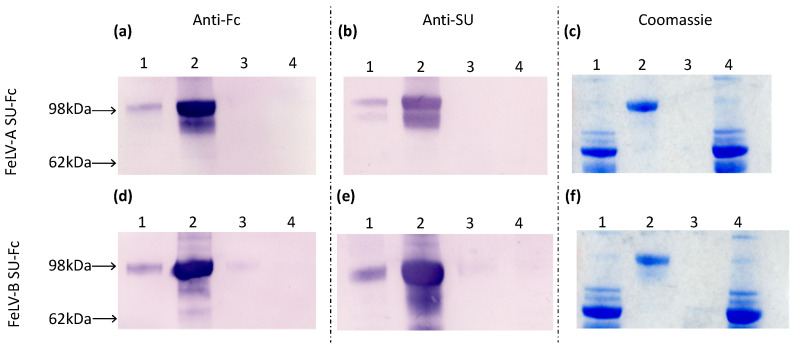
Culture fluids were collected at the following stages of protein purification: (1) pre-purification, (2) post-purification, (3) PBS wash-through and (4) ultrafiltrate waste. In immunoblot analysis of samples from the FeLV-A and FeLV-B SU purification, membranes were probed with either anti-Fc antibody (**a** and **d**, respectively) or anti-SU antibody (**b** and **e**, respectively), while gels were stained with Coomassie blue (**c** and **f**, respectively).

Samples of culture fluid collected before and after protein purification, the ultrafiltrate waste and PBS wash were analysed by immunoblotting and Coomassie staining, demonstrating the presence of FeLV-A and FeLV-B SU proteins (Figure 2). The SU-Fc proteins were detected in the pre- and post-purification fractions and were absent from the ultrafiltrate waste and PBS wash, indicating that expression and purification were successful.

### 3.2. Immunoblot Analysis of Field Samples and Samples from FeLV Naïve SPF Cats

**Figure 3:**
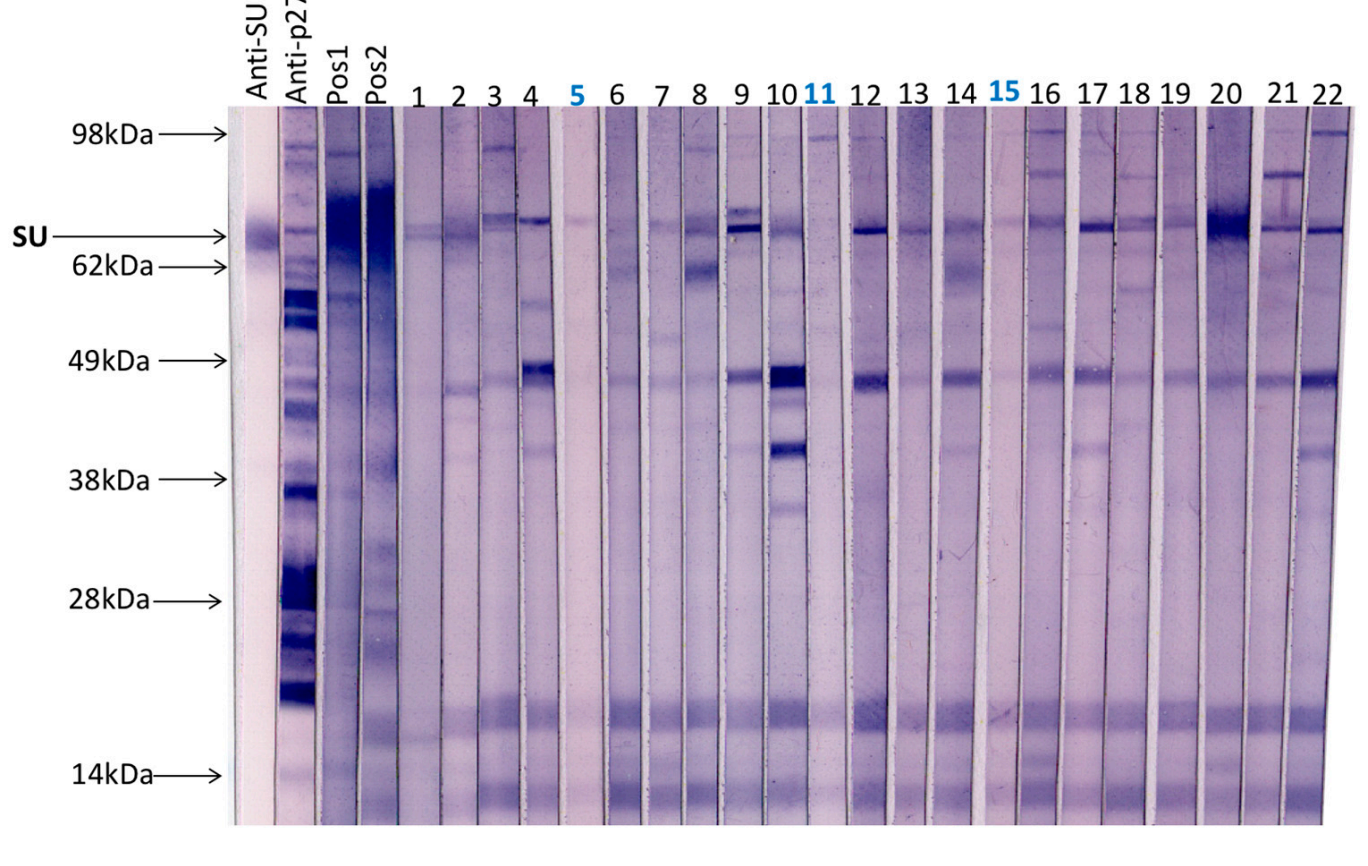
A total of 20 field cat plasma samples (samples 1–20) and two plasma samples from FeLV naïve specific pathogen free cats (samples 21 & 22) were tested by immunoblotting to determine antibody reactivity to FeLV-A proteins. Each strip was probed individually with a cat plasma or antibody control. Anti-SU (surface unit, gp70) and anti-p27 antibodies were used as controls as well as a pooled positive plasma sample with a VNA titre ≥1:128 (Pos1) and a cat plasma with a high VNA titre (VNA titre ≥1:32, Pos2). Three samples with the least reactivity to FeLV-A virus (highlighted in blue, samples 5, 11 and 15) were selected, pooled and used subsequently as a negative control.

A total of 20 field cat plasma samples (samples 1–20) and two plasma samples collected from FeLV naïve specific pathogen free cats (samples 21 & 22) were examined by immunoblotting (Figure 3). All samples had tested negative for p27 capsid antigen by IDEXX FeLV PetChek^®^ ELISA (data not shown). Immunoblots prepared using virus purified from F422 culture fluids, were cut into strips that were individually probed with cat plasma or control antibody. Three samples (5, 11 and 15) demonstrated the least reactivity against FeLV-A virus; these samples were selected, pooled and used as a negative control in subsequent SU ELISAs.

### 3.3. Assay Performance Analysis

**Figure 4:**
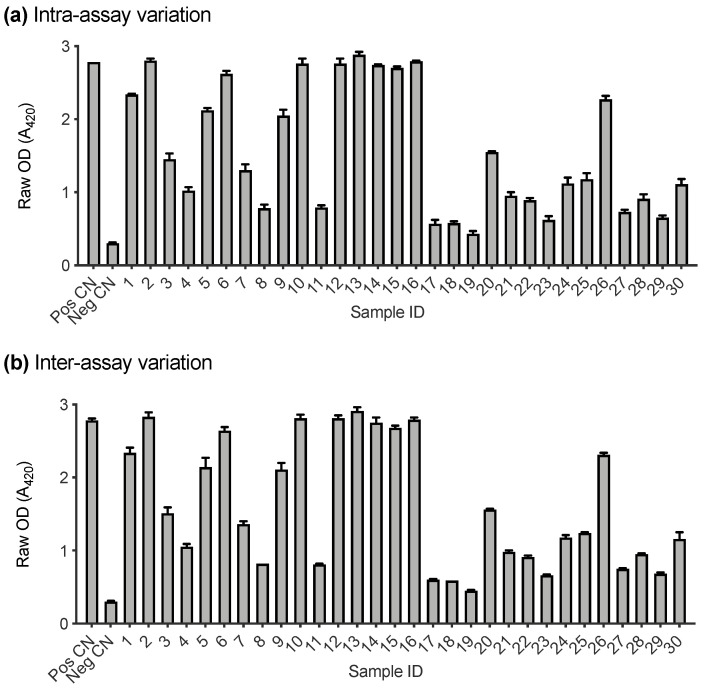
Intra- and inter-assay variation of FeLV-A SU ELISA. (**a**) Variation between samples tested in triplicate on the same plate. (**b**) Variation between samples tested on two different plates on two different days. Error bars represent standard deviation which was ≤0.13 in all cases.

A subset of samples submitted to IDEXX Laboratories (Westbrook, ME, USA) for FeLV testing were used to assess the performance of the FeLV-SU assay (*n* = 36). Firstly, the intra- and inter-assay variation of the FeLV-A SU ELISA were investigated. Positive and negative controls and 30 randomly selected cat plasma samples were tested in triplicate for antibodies recognising FeLV-A SU on two different days. Figure 4a demonstrates the intra-assay variation between replicates tested on the same plate. The error bars represent the standard deviation between replicates (which was ≤0.08 in all cases). Figure 4b demonstrates the inter-assay variation between samples tested on two different plates, processed on two different days. The error bars represent the standard deviation of the samples between plates (which was ≤0.13 in all cases).

**Figure 5:**
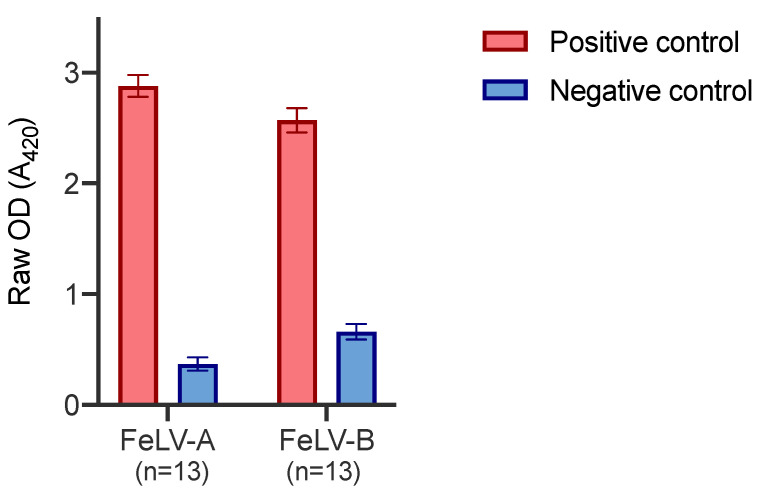
Inter-assay variation of FeLV-A and FeLV-B SU ELISA. The positive control comprised plasma collected and pooled from experimentally infected cats known to have a high VNA titre against FeLV-A. The negative control comprised pooled plasma samples that showed low reactivity to FeLV-A by immunoblot. Error bars represent standard deviation values which were ≤0.11 in all cases.

Positive and negative control plasma samples were tested on thirteen different FeLV-A SU and FeLV-B SU plates, on thirteen different days. Figure 5 demonstrates the inter-assay variation between plates. The error bars represent the standard deviation which was ≤0.11 in all cases.

**Figure 6:**
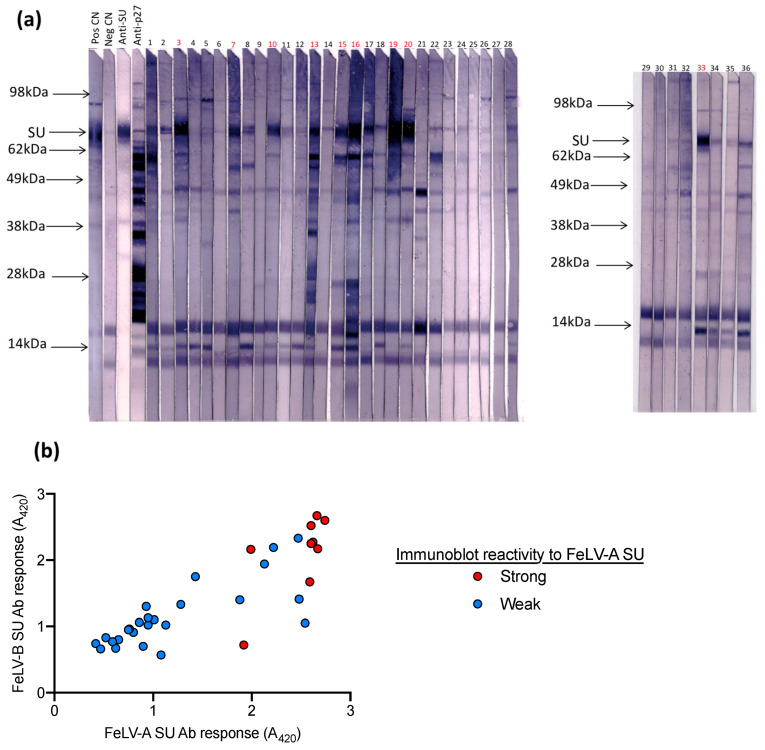
Plasma samples were tested for (**a**) antibodies against FeLV-A by immunoblot and (**b**) for antibodies against FeLV-A SU and FeLV-B SU by ELISA. Samples with high reactivity (i.e., similar to the positive control) were deemed to have a strong response to FeLV-A SU by immunoblot analysis (highlighted in red). Samples with a strong response to FeLV-A and FeLV-B SU by ELISA showed strong reactivity to FeLV-A SU by immunoblot.

Next, samples submitted to IDEXX Laboratories (Westbrook, ME, USA) for FeLV testing were tested on the FeLV-A SU ELISA, the FeLV-B SU ELISA and by immunoblot analysis against FeLV-A (*n* = 36). Samples with high antibody responses to FeLV-A SU and FeLV-B SU also demonstrated strong reactivity against FeLV-A SU by immunoblotting, as shown in Figure 6.

### 3.4. Assigning Exposure Outcomes

Exposure outcome categories were assigned according to FeLV test results. Cats with negative p27 capsid antigen, PBMC virus isolation, and PBMC proviral DNA PCR test results were classified as uninfected. Cats that tested positive for antigen but negative by PBMC virus isolation and PBMC proviral DNA PCR were classified as discordant. Cats that tested positive for PBMC proviral DNA, but were negative by PBMC virus isolation, were classified as having regressive infection, irrespective of the results of antigen testing. Cats that tested positive for PBMC proviral DNA, viral antigen and tested positive by PBMC virus isolation were classed as having progressive infection. If more than one sample was analysed from each cat, exposure outcomes were determined on a cat level and not a timepoint level. For example, some cats classified as having regressive infection tested negative for PBMC proviral DNA at timepoint 2 (TP2). However, since a positive test for PBMC proviral DNA had been recorded at timepoint 1 (TP1), such cats were determined to have regressive infection and it was considered that the proviral load was below the limit of detection at TP2. Table 3 summarises the characteristic test results for each exposure outcome, the number of cats (with a single sample and with repeated samples) assigned to each outcome and the total number of samples tested. Samples were deemed FeLV virus isolation positive only if the PBMC culture fluids tested positive for both p27 capsid antigen and RT. No cats were determined to have focal infections.

### 3.5. Antigenaemia in Different FeLV Exposure Outcome Groups

**Figure 7:**
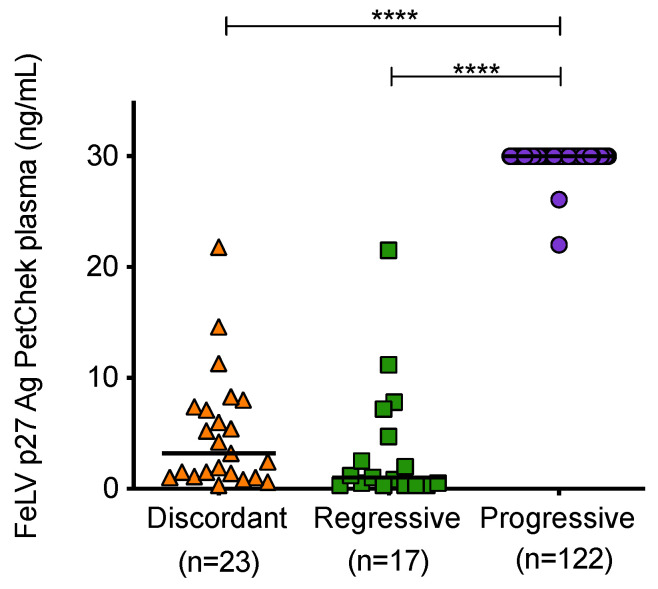
Plasma antigenaemia was measured using the IDEXX FeLV PetChek^®^ ELISA. Each point represents the p27 capsid antigen (Ag) concentration in samples from cats in the three categories. Samples from uninfected cats tested p27 capsid antigen negative. Statistical significance was determined using Kruskal–Wallis and Dunn’s multiple comparisons tests (*p*-value < 0.0001 ****).

Mean plasma p27 capsid antigen concentrations of samples from cats with different exposure outcomes were found to be significantly different (Figure 7). Samples from cats with progressive infection displayed significantly higher concentrations of p27 capsid antigen in plasma compared to samples from discordant cats and cats with regressive infection.

**Figure 8:**
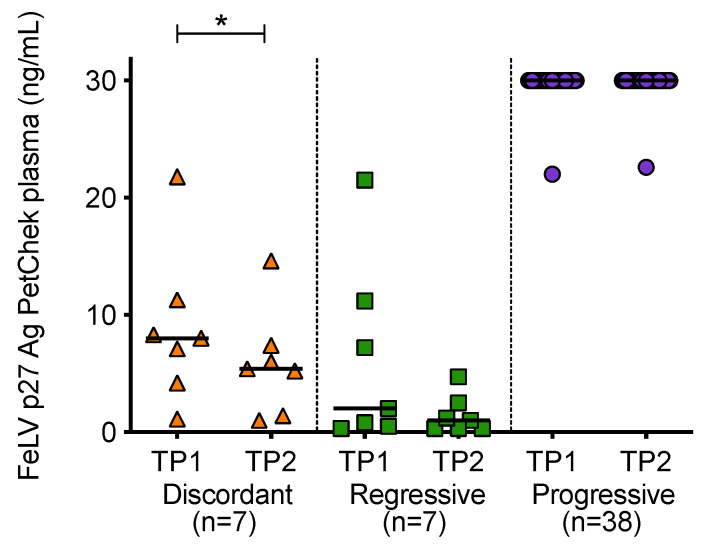
Plasma antigenaemia was measured using the IDEXX FeLV PetChek^®^ ELISA. Timepoint 1 (TP1) and timepoint 2 (TP2) p27 capsid antigen (Ag) concentrations were compared within each of the exposure outcome groups. Statistical significance was determined using Wilcoxon matched-pairs signed rank test (*p*-value < 0.05 *).

When p27 capsid antigen concentrations were compared over time, it was observed that the concentration of antigen in plasma decreased significantly between the two time points in discordant cats (Figure 8). High antigen concentrations were maintained in cats with progressive infection.

### 3.6. Proviral Load of Samples from Cats with Regressive and Progressive Infection

**Figure 9:**
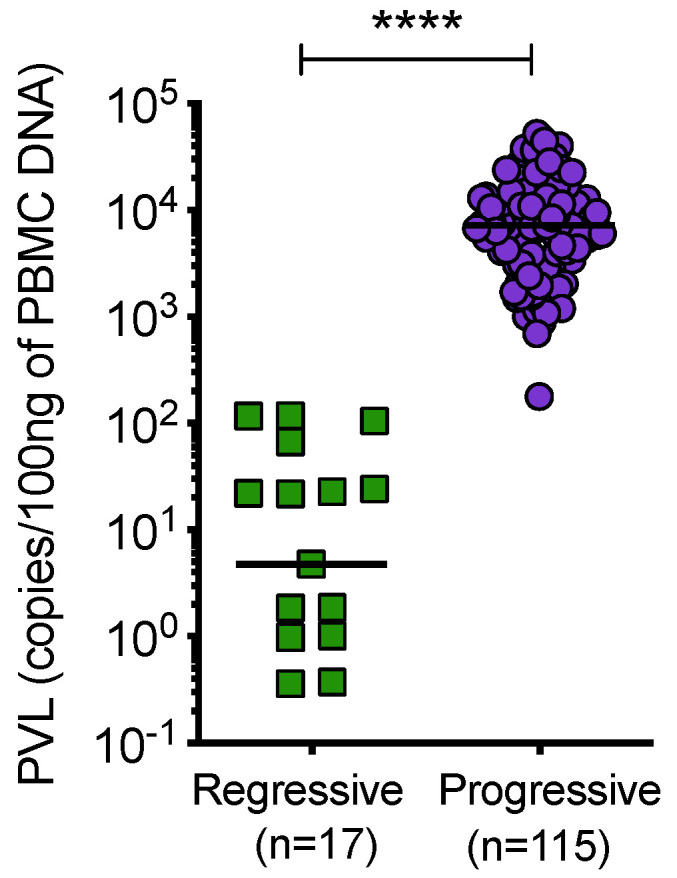
Proviral load (PVL), per 100 ng of PBMC DNA, was determined by qPCR. Cats with regressive infection showed significantly lower proviral loads compared to cats with progressive infection. Proviral load was not determined for 7 samples from cats with progressive infection. Statistical significance was determined using Mann–Whitney test (*p*-value < 0.0001 ****).

Cats with regressive infection showed significantly lower proviral loads compared to cats with progressive infection (Figure 9). Samples from uninfected and discordant cats tested negative for PBMC proviral DNA at all timepoints.

### 3.7. Analysis of PBMC Culture Fluids for p27 Capsid Antigen and RT

**Figure 10:**
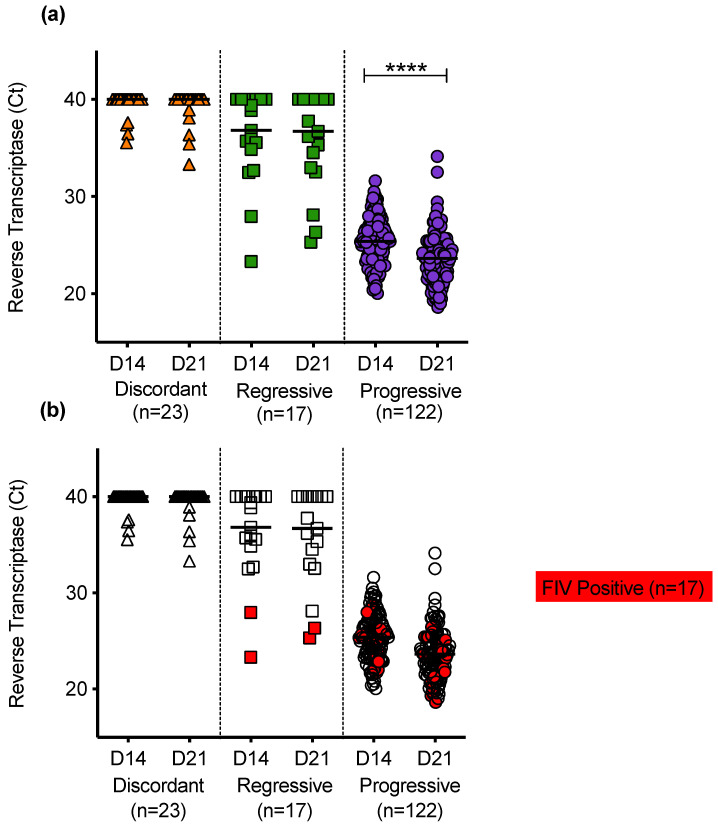
PBMC culture fluid reverse transcriptase (RT) activity in different exposure outcome groups (**a**) Detection of RT in PBMC culture fluids collected on day 14 (D14) and day 21 (D21) following in vitro culture of PBMC. No RT was detected in culture fluids from uninfected cats. (**b**) The FIV status was known for all cats tested. Samples testing positive for FIV antibodies using IDEXX SNAP^®^ FIV/FeLV Combo Test are highlighted in red. Statistical significance was determined using the Wilcoxon matched-pairs signed rank test (*p*-value < 0.0001 ****).

Reverse transcriptase was detected in PBMC culture fluids of discordant cats; cats with regressive infection; and cats with progressive infection (Figure 10a). Uninfected cats had no detectable RT in PBMC culture fluids. RT activity was generally higher in the culture fluids from cats with progressive infection. In samples from cats with progressive infection, the mean RT activity increased significantly between D14 and D21 of culture, consistent with viral replication. On D14 of culture, the PBMC culture fluids were sampled, the media were removed and cultures were replenished with fresh media and then cultured for a further 7 days. The RT detected on D21 therefore indicates continued retrovirus replication. The PERT assay detects functional RT and therefore detects all retroviruses. The FIV status was determined for all cats and cats testing positive for FIV antibodies using the IDEXX SNAP^®^ FIV/FeLV Combo Test are highlighted in red, Figure 10b.

**Figure 11:**
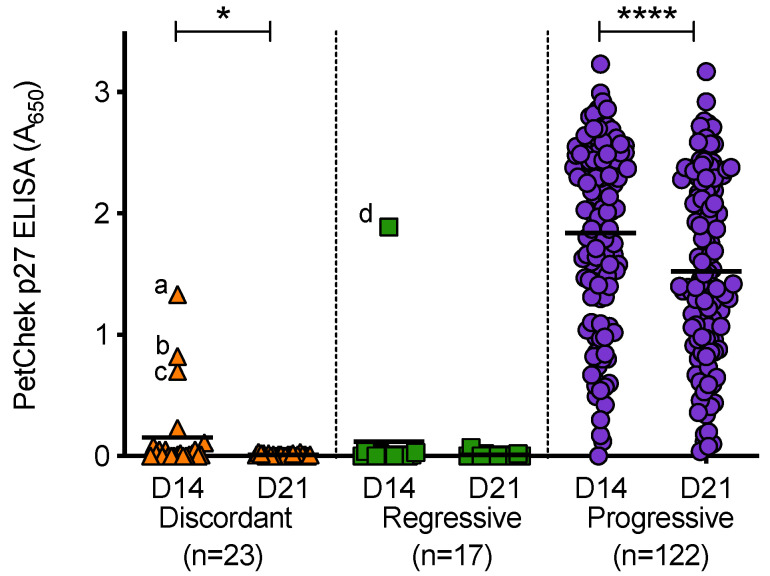
PBMC culture fluid p27 capsid antigen detection in different exposure outcome groups. PBMC culture fluids collected on day 14 (D14) and day 21 (D21) of PBMC in vitro culture were tested for p27 capsid antigen using the IDEXX PetChek^®^ ELISA and p27 antigen concentrations (absorbance values, A_650_) are shown. Samples a, b, c and d were outlier samples that tested p27 capsid antigen positive. Statistical significance was determined using Wilcoxon matched-pairs signed rank test (*p*-value < 0.05 * and < 0.0001 ****).

PBMC culture fluids (from D14 and D21 of culture) were tested for p27 capsid antigen, Figure 11. PBMC culture fluids from uninfected cats tested negative for p27 capsid antigen. All PBMC culture fluid samples from discordant cats and cats with regressive infection tested negative for p27 capsid antigen; with the exception of three D14 discordant PBMC culture fluid samples and one D14 PBMC culture fluid sample from a cat with regressive infection. A range of p27 capsid antigen concentrations were detected in PBMC culture fluids from cats with progressive infection (absorbance values at 650 nm ranged from 0 to 3.23). Not all PBMC culture fluid samples from cats with progressive infection contained detectable levels of p27 capsid antigen. In general, the concentrations of p27 capsid antigen in D21 culture fluids were lower than those measured in D14 culture fluids (the converse was true for RT detection). This decrease was statistically significant in the discordant and progressive outcome groups. All of the D21 culture fluids that tested positive were confirmed using the IDEXX FeLV PetChek^®^ ELISA neutralisation protocol, as were outlier samples (denoted as a, b, c and d). Outlier samples ‘b’ and ‘d’ also tested positive for RT.

### 3.8. Humoral Immune Response of Cats with Different FeLV Exposure Outcomes

**Figure 12:**
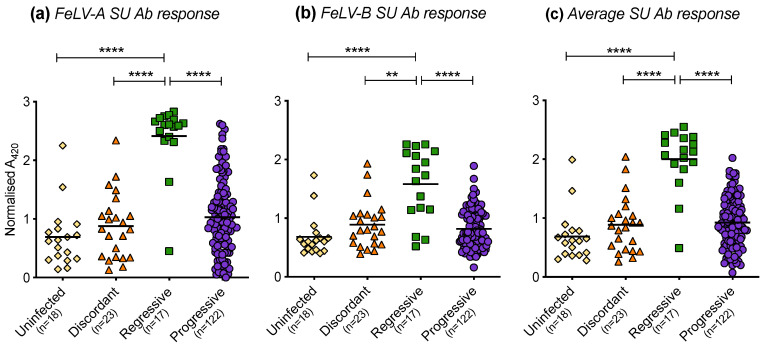
FeLV-A, FeLV-B and average SU antibody responses in different exposure outcome groups. Plasma samples were tested for antibodies recognising FeLV-A SU (**a**) and FeLV-B SU (**b**) proteins and the average SU antibody (Ab) responses are shown (**c**). Statistical significance was determined using Kruskal–Wallis and Dunn’s multiple comparisons tests (*p*-value < 0.01 ** and < 0.0001 ****).

The humoral immune responses of cats with different FeLV exposure outcomes were found to differ significantly. Cats with regressive infection showed the highest antibody responses to FeLV-A (Figure 12a) and FeLV-B SU (Figure 12b). The FeLV-A and FeLV-B SU antibody responses of discordant cats were similar to those of uninfected cats and cats with progressive infection. The average anti-FeLV SU antibody responses were calculated from the FeLV-A and FeLV-B SU antibody responses, Figure 12c. Statistically significant differences were observed amongst cats with different exposure outcomes. Cats with regressive infection showed significantly higher average antibody responses to FeLV-SU proteins compared to cats in other exposure outcome groups. Cats with progressive infection displayed similar average antibody responses to FeLV-SU proteins to uninfected cats and discordant cats.

**Figure 13:**
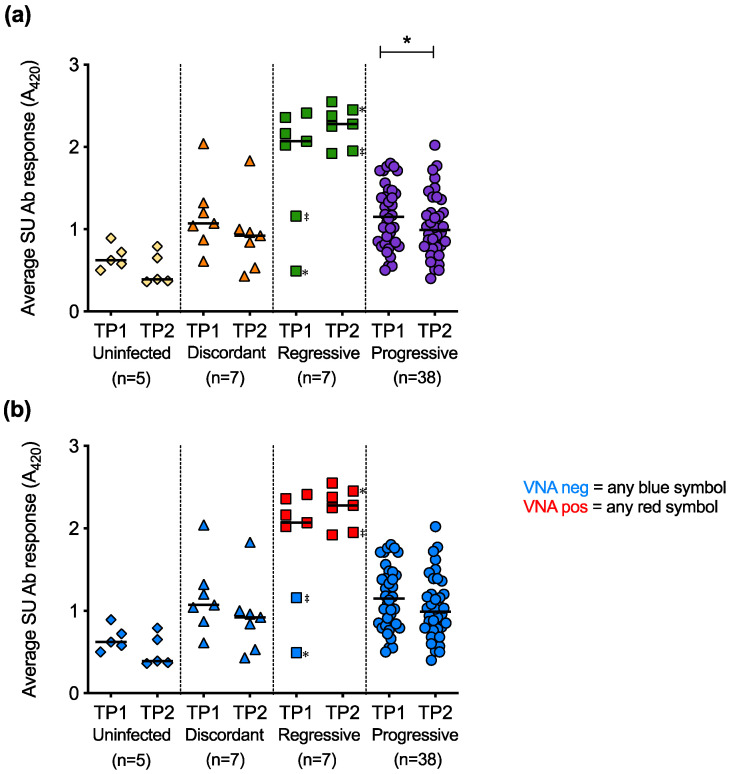
Longitudinal analysis of average SU antibody responses and VNA responses. (**a**) For paired samples, average anti-SU responses calculated at timepoint 1 (TP1) and timepoint 2 (TP2) are shown. (**b**) All plasma samples were then tested for virus neutralising antibodies (VNA). Two cats with regressive infection (‡ and ✻) seroconverted from negative to positive between TP1 and TP2. Statistical significance was determined using Wilcoxon matched-pairs signed rank test (*p*-value < 0.05 *).

The average SU antibody responses at TP1 were compared to the average SU antibody responses at TP2 for longitudinal samples (Figure 13a). The decrease in SU antibody responses over time was statistically significant in the cats with progressive infection. Live virus neutralisation assays were performed to determine whether the antibodies against FeLV SU detected by ELISA were neutralising (Figure 13b). Only plasma samples from cats with regressive infection tested positive for virus neutralising antibodies (VNA). Two cats with regressive infection that had low antibody responses to FeLV-A and FeLV-B SU proteins at TP1 showed high antibody responses to the proteins at TP2. This increase in antibody response to the SU proteins was accompanied by the detection of VNA at TP2, consistent with seroconversion.

**Figure 14:**
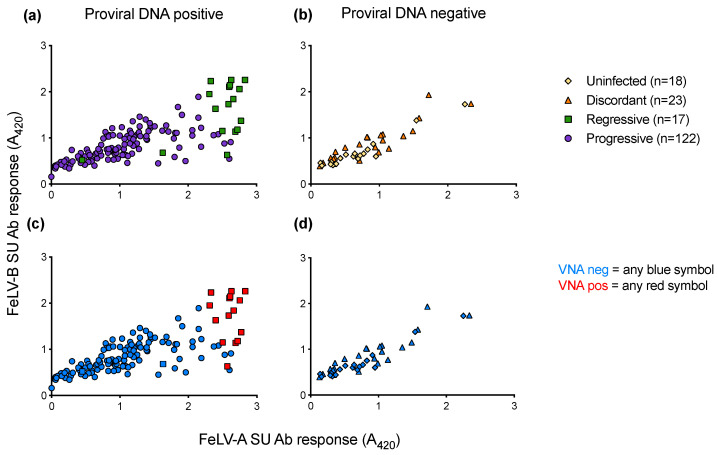
Antibody responses to FeLV-A and FeLV-B SU proteins and VNA responses. Plasma samples were tested for anti-FeLV-A SU and anti-FeLV-B SU antibodies by ELISA. The antibody response to FeLV-A SU is shown on the x-axis, with the antibody response to FeLV-B SU on the y-axis. (**a**) SU antibody responses of PBMC proviral DNA positive samples. (**b**) SU antibody responses of PBMC proviral DNA negative samples. Plasma samples were tested using live virus neutralisation assays to detect VNA (**c**) VNA results of PBMC proviral DNA positive samples. (**d**) VNA results of PBMC proviral DNA negative samples. Only cats with regressive infection displayed VNA (red).

When anti-FeLV-A SU antibody responses were compared to anti-FeLV-B SU antibody responses, it was observed that samples that tested positive for VNA clustered together, demonstrating high antibody responses to both SU proteins (Figure 14). VNA titres were detected in 15 of the 17 samples from cats with regressive infection. All VNA-positive cats showed VNA titres of ≥1:32.

## 4. Discussion

This study provided a unique opportunity to analyse the antibody responses of cats naturally exposed to FeLV. The SU ELISA is a novel assay that was developed to assess the immune response to the SU proteins of FeLV. Previously, a FeLV SU ELISA was used to measure antibody responses in naturally infected cats, using p45, non-glycosylated gp70 as the antigen [34]. In addition, antibody responses to p45 were studied by Boenzil et al. [35], although it was concluded that testing for antibodies recognising p15E had greater diagnostic potential. In this study, by expressing the FeLV SU proteins in mammalian cell culture as Fc-tagged fusion proteins, it was possible to generate sufficient protein, of the required purity, for use in a new ELISA. Moreover, following purification, the proteins retained antigenicity and were recognised by sera containing VNA. Minimal inter-and intra-assay variation was observed, indicating that this assay was suitable for the assessment of the immunocompetence of test subjects. Plasma samples were screened for anti-FeLV-A and anti-FeLV-B SU antibodies to ensure a comprehensive analysis of the antibody response to SU. Immunoblotting was used to assess the antibody reactivity of field samples and FeLV naïve SPF samples against FeLV-SU; samples with negligible responses were selected, pooled and used as a negative control. A range of samples were tested for antibodies against FeLV-A SU and FeLV-B SU using the ELISA described here as well as for antibodies recognising FeLV-A SU following immunoblot analysis. Samples showing a high response on the ELISA also showed strong reactivity to the SU protein on immunoblot analysis. Not all samples with a high response to the SU proteins on the ELISA demonstrated strong reactivity to FeLV-A SU by immunoblot. It is likely that the ELISA detected samples with antibodies recognising conformational and not linear epitopes on FeLV SU. It was not possible to estimate ELISA cut-off values or the sensitivity and specificity of the assay as insufficient numbers of samples from cats of known FeLV serostatus were available. As the samples used in this study came from stray cats submitted to the Austin Pets Alive! Shelter, it was not possible to determine whether the cats had been previously vaccinated. Given that the cats were strays, it is highly unlikely that any of the cats had been vaccinated against FeLV, however, we cannot exclude this possibility. None of the cats were vaccinated by the shelter during the study.

The terminology for FeLV exposure outcomes has been discussed widely in the literature. However, in order to draw comparisons between cats with different test results, exposure outcome categories were assigned in this study, based on antigenaemia, PBMC virus isolation and the detection of PBMC proviral DNA. The majority of cats tested had progressive infection (68.3%). Cats with progressive infection displayed high proviral loads, were antigenaemic and tested positive following PBMC virus isolation. This is a consistent finding, and it has been suggested that higher proviral loads in cats with progressive infection is associated with a poorer prognosis [5,36]. From these findings we concluded that the identification of viraemic animals, those posing a risk of transmission to other cats, is more readily achieved than the identification of cats in the other outcome groups. Cats with abortive infection have been reported to display no evidence of FeLV infection, with the possible exception of detectable antibodies [5,13]. In this study, only cats with regressive infection displayed VNA and therefore no cats were assigned to the abortive infection category. However, two uninfected cats displayed high antibody responses to both SU proteins in spite of testing negative for VNA, suggesting that these cats could have had abortive infection or might have been vaccinated previously against FeLV. Discordancy, whereby cats tested positive for p27 capsid antigen by ELISA but tested negative for either PBMC proviral DNA by qPCR or cell-free virus by PBMC virus isolation, was observed in 13% of cats (16/123). Of the 16 discordant cats, follow-up samples were received from 7 cats. Antigenaemia persisted in these discordant cases despite the cats remaining negative for PBMC proviral DNA. FeLV proviral DNA could not be detected in any of the PBMC DNA samples from the discordant cats, suggesting that there had been no bone marrow infection. Naturally occurring focal infections have been documented rarely [15], but it is possible that the discordant cats identified in this study had focal infections and focal infection might be more common than previously estimated. This hypothesis warrants further investigation. Some of the discordant cats, and cats with regressive infection, were antigenaemic. Indeed, the concentrations of p27 capsid antigen were statistically higher in cats with progressive infection compared to other exposure outcome groups. As expected, follow-up samples revealed that antigenaemia levels decreased over time in discordant cats. Conversely, high plasma antigenaemia levels were maintained throughout progressive infection, indicating that quantitative, sequential antigenaemia testing is beneficial in FeLV diagnostic testing. Since the assay used to measure antigenaemia had an upper detection limit of 30 ng/mL, it is possible that some cats with progressive infection could have had antigen levels greater than 30 ng/mL at both timepoints.

Reverse transcriptase activity was detected in the PBMC culture fluids of discordant cats, cats with regressive infection and cats with progressive infection. The PERT assay detected enzymatically active reverse transcriptase and hence confirmed the presence of any retroviruses in the cultures. All cats were tested for FIV antibodies using IDEXX SNAP^®^ FIV/FeLV Combo Test and some, but not all, of the positive results from cats with regressive infection could be explained by FIV co-infection. All PBMC culture fluids from uninfected cats tested negative for RT activity, suggesting low level FeLV infection could have been the source of the RT activity detected in the PBMC culture fluids from cats with other exposure outcomes. However, the suspected low level FeLV infection was not confirmed; when culture fluids were tested for p27 capsid antigen, the majority tested negative. The detection of RT activity in the absence of FeLV p27 capsid antigen in these cats could indicate the presence of another retrovirus, such as feline foamy virus (FFV), although this was not confirmed. PBMC culture media were replenished on day 14 of culture after sampling. Therefore, any RT or p27 antigen present in the culture fluid on day 21 of culture represents the continued production of virus from these cultures. PBMC cultures from discordant cats and cats with regressive infection that tested positive for RT at D14 maintained RT production to D21. The four outlier PBMC culture fluid samples that tested positive for p27 capsid antigen on day 14 (a, b, c and d) tested negative on day 21, suggesting that the low level of FeLV infection in these cultures was not sustained beyond day 14.

Uninfected cats tested antigen negative, PBMC proviral DNA negative, PBMC virus isolation negative and had low antibody responses to the SU proteins. It was assumed that the majority of the cats in this outcome group had not been exposed to FeLV, with the possible exception of two outlier cats that exhibited higher antibody responses compared to the rest of the group. Discordant cats also exhibited low antibody responses to the SU proteins. If these cats had focal infection, the level of humoral immunity might depend on the tissues in which the virus was replicating. Cats with regressive infection had the highest mean antibody responses to FeLV-A and FeLV-B SU proteins, correlating with lower proviral loads and potentially better prognoses. Cats with progressive infection showed significantly lower antibody responses to the SU proteins compared to cats with regressive infection, correlating with higher proviral loads and potentially poorer prognoses [36].

The mean SU antibody response was calculated at TP1 and TP2. A statistically significant decrease in mean SU antibody response was observed between the two timepoints in cats with progressive infection. In cats with poor immune responses to infection, viral replication likely proceeds unhindered, leading to FeLV-induced immunosuppression and a decrease in immune function. The majority of the cats with regressive infection maintained high responses between TP1 and TP2. The maintenance of an effective immune response in cats with regressive infection is indicative of viral control, resulting in a good prognosis. Two cats with regressive infection showed evidence of seroconversion from negative to positive between TP1 and TP2; the first sample from each cat displayed low antibody responses to both SU proteins and tested negative for VNA whereas the second sample from each cat had high antibody responses to both SU proteins and contained VNA. This emphasises the importance of sample timing and follow-up sampling [18]. Nevertheless, both cats showed high Ct values when tested by FeLV qPCR at TP1 (≥Ct 28) and low amounts of RT activity were detected in PBMC culture fluids. These parameters are consistent with regressive infection, in spite of the low antibody responses to SU and lack of VNA. With the exception of the first samples from these two cats, all samples from cats with regressive infection contained VNA and only cats with regressive infection had detectable VNA titres in this study. Cats that tested VNA positive had high responses to both FeLV-A and FeLV-B SU proteins. One potential explanation for these results is that an antibody response to an epitope shared between FeLV-A (Glasgow) and FeLV-B (Gardner-Arnstein) confers neutralisation, but this hypothesis requires further investigation.

When comparing the proviral loads and SU antibody responses of cats with regressive and progressive infection, these parameters clearly segregated into two separate groups. Cats with regressive infection had higher SU antibody responses (associated with better infection outcomes) and lower proviral loads compared to cats with progressive infection (associated with worse infection outcomes). Cats with discordant results might have had highly suppressed regressive or focal infections, such that they tested PCR negative. The cellular immune response was not measured in this study; however, it is known to play a role in FeLV recovery [22] and could be facilitating recovery from viraemia in cats where antibodies and VNA were not detected. The FeLV SU ELISA responses did not correlate with exposure, as cats with progressive infection had been exposed to FeLV and yet showed weak antibody responses against FeLV-SU. Therefore, this assay could not be used to determine FeLV exposure history. Rather, higher antibody responses against the FeLV-SU proteins were indicative of an effective immune response following FeLV infection and could be used alongside other FeLV diagnostic tests to provide prognostic information to the clinician.

## 5. Conclusions

The immune response to FeLV is complex and predicting the outcome of FeLV infection in an infected animal remains challenging, despite the abundance of diagnostic tests available. This study aimed to improve our understanding of the pathogenesis of FeLV infection by investigating how best to interpret diagnostic tests to assess disease progression and determine prognosis. Cats with regressive infection had higher anti-SU antibody responses and VNA titres, as well as lower p27 capsid antigen levels and lower PBMC proviral DNA loads compared to cats with progressive infection. The data generated in this study provides evidence that measuring anti-SU antibody responses as well as the p27 capsid antigen concentration and proviral load might improve FeLV diagnostics, since the viral burden and the immune status can be assessed by the clinician to provide prognostic information. The FeLV SU ELISA described here has been used to demonstrate active immune control in a subset of cats with low levels of p27 capsid antigen [37] that were assumed to have regressive infection as well as to investigate correlates of protection following FeLV vaccination [38].

## Figures and Tables

**Table 1:** Diagnostic test results for each exposure outcome and test turnaround time.

Test Turnaround Time	Hours	Hours	1 Week	5 Days
Outcome of Exposure	p27 Capsid Antigen	Proviral DNA	Plasma Virus Isolation	Virus Neutralising Antibody
Abortive infection	Negative	Negative	Negative	Positive
Regressive infection (shedding)	Positive	Positive	Positive	Negative
Regressive infection (recovered)	Negative	Positive	Negative	Positive
Progressive infection	Positive	Positive	Positive	Negative
Focal infection	Positive	Positive (*)	Negative	Positive

(*) Infected tissues from cats with focal infection will test proviral DNA positive. Bone marrow and blood from these cats will test negative for proviral DNA.

**Table 2:** Number of months between collection times of paired samples.

Months between Timepoints	Number of Cats
1	1
2	0
3	5
4	5
5	3
6	43

**Table 3:** Exposure outcome categories were assigned based on p27 capsid antigen, PBMC virus isolation and PCR results.

Outcome of Exposure	p27 Capsid Antigen	PBMC Proviral DNA	PBMC Virus Isolation	Total No. of Cats	No. of Cats with Single Sample	No. of Cats with Two Samples	Total No. of Samples
Uninfected	Negative	Negative	Negative	13	8	5	18
Discordant	Positive	Negative	Negative	16	9	7	23
Regressive	Positive/Negative	Positive (*)	Negative	10	3	7	17
Progressive	Positive	Positive	Positive	84	46	38	122

(*) Cats testing PBMC virus isolation negative but PBMC proviral DNA positive on at least one time point were classified as having regressive infection.

## Data Availability

Data generated using the IDEXX FeLV PetChek^®^ ELISA is available on request due to restrictions. The data are not publicly available due to this being a proprietary commercial assay.
